# Peripheral ischaemic retinopathy and neovascularisation in a patient with subacute streptococcus mitis-induced bacterial endocarditis

**DOI:** 10.3205/oc000076

**Published:** 2017-09-12

**Authors:** Laura S. Leysen, Elke O. Kreps, Ilse De Schryver, Kristien P. Hoornaert, Vanessa Smith, Julie De Zaeytijd

**Affiliations:** 1 Department of Ophthalmology, Ghent University Hospital, Ghent, Belgium; 2 Department of Rheumatology, Ghent University Hospital, Ghent, Belgium

**Keywords:** retinal diseases, retinal neovascularisation, subacute bacterial endocarditis

## Abstract

**Objective:** To describe a patient with peripheral retinal ischaemia and neovascularisation who was diagnosed with streptococcus mitis-induced bacterial endocarditis.

**Methods:** Retrospective analysis of case report. A 57-year-old man presented with a history of a rapidly progressive, bilateral, painless visual loss. He also suffered from pain in the neck and lower back and a weight loss of 10 kg. He underwent a full ophthalmologic work-up, laboratory investigations, and imaging of the spine.

**Results:** BCVA was reduced to 20/40 in the right eye and 20/32 in the left eye. Fundoscopy showed rare intra-retinal haemorrhages including few Roth spots and cotton wool lesions. Fluorescein angiography demonstrated large areas of peripheral retinal ischaemia and neovascularisation. Imaging of the spine showed spondylodiscitis on several levels. Further imaging and blood cultures confirmed bacterial endocarditis of the mitral valve. Streptococcus mitis was subsequently identified as the causative organism.

**Conclusion:** Peripheral retinal ischaemia and neovascularisation were previously unrecognised as a feature of infectious endocarditis. Therefore, their presence, apart from the classic Roth spots, should prompt the consideration of infectious endocarditis in the etiologic work-up.

## Introduction

Peripheral retinal neovascularisation may result from several underlying systemic and ocular conditions, such as hyperviscosity syndromes, sarcoidosis, sickle cell retinopathy, vasculitis, pars planitis, branch and central retinal vein occlusion, ocular ischaemic syndrome, Eales disease, longstanding retinal detachment, and talc embolisation [1], [2], [3]. The prototype of peripheral neovascularisation is proliferative diabetic retinopathy, resulting from capillary occlusions due to chronic hyperglycemia. Amongst these entities, hyperviscosity syndromes stands out as a condition that can cause both peripheral retinal occlusion and Roth spots [2], [4]. To our knowledge, subacute bacterial endocarditis has not been described as a cause of these combined ocular features.

## Case description

**Figure 1:**
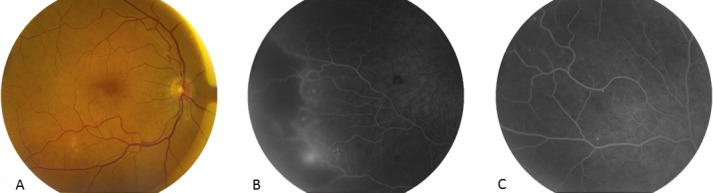
RE. A) Macula with cotton wool spots and minor microangiopathy signs temporal from the fovea; B) FFA temporal periphery highlighting an area of ischemia bordered by neovascularisation; C) FFA superior with microangiopathy

**Figure 2:**
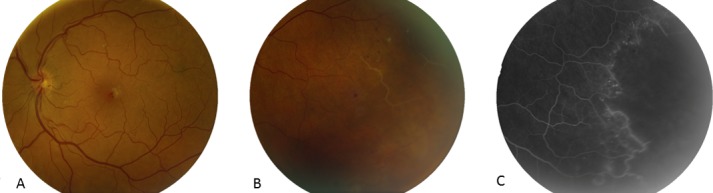
LE. A) Macula with hemorrhage and embolisation at the fovea; B,C) temporal periphery demonstrating occluded vessels with corresponding area on FFA

A 57-year-old man presented with a history of a rapidly progressive, bilateral, painless visual loss ongoing for two weeks. He also suffered from pain in the neck and lower back and had a weight loss of 10 kg. He underwent full ophthalmologic work-up. At presentation, BCVA was reduced to 20/40 in the right eye and 20/32 in the left eye. Slit-lamp examination was unremarkable. On fundoscopy, we noticed scattered intra-retinal haemorrhages including a few Roth spots and cotton wool lesions (Figure 1A (Fig. 1), Figure 2A,B (Fig. 2)). Fluorescein angiography revealed large areas of temporal peripheral retinal ischaemia in both eyes and neovascularisation in the right eye (Figure 1B,C (Fig. 1), Figure 2C (Fig. 2)). General history was significant for diabetes mellitus type 2 (since 15 years) and low-grade mitral valve insufficiency. The current white-centred haemorrhages together with the peripheral vascular occlusions prompted systemic investigations, primarily in search for hyperviscosity syndromes and inflammatory markers. Laboratory tests demonstrated elevated CRP of 38.8 mg/L (ref <10 mg/L) and erythrocyte sedimentation rate of 51 mm/hour (ref 4–8 mm/hour) and otherwise normal blood counts, protein electrophoresis, liver and renal function. The pain in the neck and lower back was further evaluated with bone scintigraphy revealing inflammatory hot spots scattered in both the cervical and lumbar area. An additional MRI of the spine showed both cervical and lumbar spondylodiscitis on multiple levels. This, together with raised inflammatory markers in the blood and the ocular findings guided the search towards an embolic origin. The patient underwent urgent blood cultures and ultrasonic imaging of the heart, which demonstrated mitral valve vegetations. Streptococcus mitis could be identified as causative organism for this subacute bacterial endocarditis. Treatment with ampicillin and gentamycin was started and he subsequently underwent urgent cardiac valve replacement. Unfortunately, our patient persistently refused further ophthalmologic treatment and follow-up due to general weakness and fatigue.

## Discussion

Proliferative diabetic retinopathy is the prototype of aberrant retinal neovascularisation. It is characterised by new vessel growth of the optic disc, retina and/or iris in an attempt to alleviate retinal hypoxia in areas of severe retinal capillary closure and ischemia. Our patient was known to have diabetes type 2. However, an ophthalmic review 2 months prior to presentation had disclosed no signs of diabetic retinopathy. Evolution from no to proliferative diabetic retinopathy on such a short notice is impossible in the absence of another disease. Therefore, diabetes mellitus was immediately excluded as a cause of the peripheral retinopathy in this patient. 

Peripheral retinal neovascularisation is not unique to proliferative diabetic retinopathy and may occur in a host of systemic and purely ocular disease processes. The differential diagnosis lists retinal vein occlusion, Eales disease, pars planitis syndrome, radiation retinopathy, and talc embolisation. Systemic diseases to be considered include hyperviscosity syndromes, (chronic) leukaemia, rheumatic fever, sarcoidosis, and sickle-cell retinopathy. A diagnostic work-up should involve a detailed history to detect previous and current systemic complaints guiding blood tests towards identification of the underlying disease.

Roth spot are white-centred retinal haemorrhages. The pale centre can be composed of coagulated fibrin including platelets, focal ischemia, neoplastic or inflammatory cells, or infectious organisms. Historically, Roth spots were considered to be pathognomonic for subacute bacterial endocarditis [4]. However, they are actually nonspecific and may occur in a number of entities, such as blood dyscrasias, hypertensive retinopathy, diabetic retinopathy, pernicious anaemia, HIV infection, and pre-eclampsia [4], [5]. Again, a detailed history together with standard blood tests can guide towards the underlying diagnosis. 

Laboratory tests in our patient demonstrated elevated inflammatory markers and otherwise normal blood counts and protein electrophoresis, prompting an in-depth evaluation of the pain in the neck and lower back. Although back pain can be caused by numerous conditions, imaging was quite distinctive for an underlying spondylodiscitis with an endocarditis (mitral valve insufficiency) as primary infection site. 

In bacterial endocarditis, microembolisation of bacteria or debris from the damaged valve frequently occurs in the brain, spleen, kidney, and skin [6]. Seldomly, other organs may show evidence of embolic involvement, including the eye as evidenced by rare cases of central retinal artery occlusion [7], [8]. In our patient, continuous metastatic microembolisation of bacteria or debris from the damaged valve uniquely led to occlusion of the peripheral retinal vessels, resulting in extensive areas of ischemia triggering a proliferative ischaemic vasculopathy. 

Peripheral retinal ischaemia and neovascularisation should be treated with argon laser photocoagulation to avoid late complications such as vitreous haemorrhage, iris neovascularisation, and neovascular glaucoma … with subsequent visual loss.

Treatment for bacterial endocarditis includes antibiotics, which can be specified according to blood cultures to identify the causative bacteria. General work-up and treatment however should be performed in collaboration with specialised departments.

## Conclusion

Peripheral retinal ischaemia and more specifically neovascularisation were previously unrecognised as a feature of infectious endocarditis. The presence of peripheral retinal ischaemia and neovascularisation, apart from the classic Roth spots should prompt the consideration of infectious endocarditis, among other differential diagnosis, in the etiologic work-up. 

## Notes

### Competing interests

The authors declare that they have no competing interests.
